# Marine resources and their value in Kadavu, Fiji

**DOI:** 10.1007/s13280-022-01794-0

**Published:** 2022-10-01

**Authors:** Simon Harding, Kalisiana Marama, Annette Breckwoldt, Ulamila Matairakula, Elodie Fache

**Affiliations:** 1 Institute of Marine Resources, The University of the South Pacific, Laucala Campus, Private Mailbag, Suva, Fiji; 2 Social-Ecological Systems Analysis, Social Science Department, Leibniz Centre for Tropical Marine Research (ZMT), Fahrenheitstrasse 6, 28359 Bremen, Germany; 3 UMR SENS, IRD, CIRAD, Univ Paul Valery Montpellier 3, Univ Montpellier, Site St Charles 2, Route de Mende, 34199 Montpellier Cedex 5, France; 4 Plumpton Green, Lewes, East Sussex UK; 5 Pacific Blue Foundation, Suva, Fiji; 6 Graduate School of Agriculture, Forestry and Fisheries, Shimoarata Campus, Kagoshima University, Kagoshima, Japan

**Keywords:** Coastal fisheries, Economic and sociocultural value, Fiji, Interdisciplinary, Marine resources, South-western Pacific

## Abstract

Recognition of the multiple types of value of marine resources is crucial to help design locally meaningful and sustainable management approaches for marine and coastal habitats. There is a lack of information on the amount of living marine resources harvested by coastal communities in many Pacific Island Countries and Territories (PICTs), as well as on their economic and non-economic value. This paper explores the monetary, subsistence, and sociocultural value of selected marine resources (finfish and invertebrates) in Kadavu province, Fiji, based on a household survey and semi-structured interviews conducted in 2019 within one specific district. The paper provides estimates of the annual catch and monetary value of marine resources harvested by local communities at both the district and provincial levels, derived from catch and effort information collected from fishers and gleaners in situ. It also highlights the importance of integrating the sociocultural significance of marine resources into future value assessments.

**Supplementary Information:**

The online version contains supplementary material available at 10.1007/s13280-022-01794-0.

## Introduction

Fish—both finfish and invertebrates—are widely recognized as a cornerstone of food, health, livelihood, and ecological security in Pacific Island countries and territories (PICTs) (Bell et al. 2009; Gillett 2016). These living marine resources provide 50–90% of animal protein for coastal communities and a very high proportion of cash and non-cash income, plus countless other ecosystem goods and services in many PICTs (Thaman et al. 2014; Bell et al. 2018). Across the western Pacific, per capita fish consumption is substantially higher than the global average, and in some of the atoll nations is among the highest in the world (Hanich et al. 2018). Inshore fisheries also provide “the primary or secondary source of income for up to 50% of households in PICTs” (SPC 2015). Most of this fish has traditionally come from small-scale coastal fisheries, which have contributed to food security both directly through subsistence fishing and indirectly through incomes earned from artisanal fishing (Bell et al. 2018).

Small-scale coastal fisheries are not only vitally important at the community level for food security and income generation, but also because they carry significant sociocultural value (e.g., Foale et al. 2011; Gordon 2013; Veitayaki et al. 2014; Fache and Pauwels 2020). In particular, some target fish can be categorized as ‘cultural keystone species’ (CKS), i.e., as “culturally salient species” that play a major role in the diet, economy, technology, medicine, narratives, ceremonies, and/or spiritual practices of a specific group of people, of which they contribute to “shape in a major way the cultural identity” (Garibaldi and Turner 2004, p. 4). For instance, salmon (*Oncorhynchus* spp.) has been described as a prime example of CKS in North America, murray cod (*Maccullochella peelii*) for Australian Aboriginal communities living within the Murray-Darling Basin, and whitebait (*Galaxias* spp.) for Māori people in Aotearoa—New Zealand (Noble et al. 2016). In Fiji, CKS include totems, chiefly foods, dietary staples, and species of high commercial and/or subsistence importance, such as trevally (*Caranx* spp.), whose documentation is lacking yet necessary as some fisheries management or biodiversity conservation policies might affect access to these species by the communities that value them (Kitolelei et al. 2021).

Recognition of the multiple types of value of marine resources is therefore crucial to help design sustainable management approaches for marine and coastal habitats that are meaningful and deemed equitable at the local level. However, there is a lack of information of the amount and monetary value of living marine resources harvested by coastal communities in many PICTs (Hanich et al. 2018). Coastal fisheries are often under-reported in PICTs (Zeller et al. 2015), and their actual economic value is usually not on the radar of national decision makers that tend to pay more attention to offshore pelagic fisheries (Gillett 2014; FAO 2017). A lack of economic information has contributed to coastal fisheries management policies being neglected at the ministerial level (Hanich et al. 2018). Many PICTs also still do not have the capacity to monitor small-scale fisheries catch adequately (Batista et al. 2014). In remote, data-poor fisheries, “surveys asking fishers to recall their catch and effort are often the only practicable method” (Barnes-Mauthe et al. 2013). Moreover, beyond its economic value, often articulated or rather opposed to an ‘intrinsic’ biodiversity value (Foale et al. 2016), the sociocultural significance of marine life for Pacific coastal communities remains often overlooked (Fache and Pauwels 2020), as does the core role and contributions of women to small-scale coastal fisheries (Waqairatu-Waqainabete 2019; Kitolelei et al. 2021; Thomas et al. 2021).

This paper explores the importance of the economic and sociocultural values of selected living marine resources (finfish and invertebrates) in a specific context in the south-western Pacific, namely for Nakasaleka district of Kadavu province in Fiji. It then discusses some implications of this assessment for coastal fisheries management at the local, provincial, and national level. In particular, it confirms that a socioeconomic approach based on household surveys can be used to (a) complement wherever possible the information retrieved by direct monitoring of small-scale coastal fisheries catch, and (b) estimate the annual harvest of marine resources as well as both their subsistence and monetary value. In addition, the paper calls for greater attention to the inter-relationships between this utilitarian value and the local sociocultural significance of marine resources.

## Materials and methods

### Study site

**Fig. 1:**
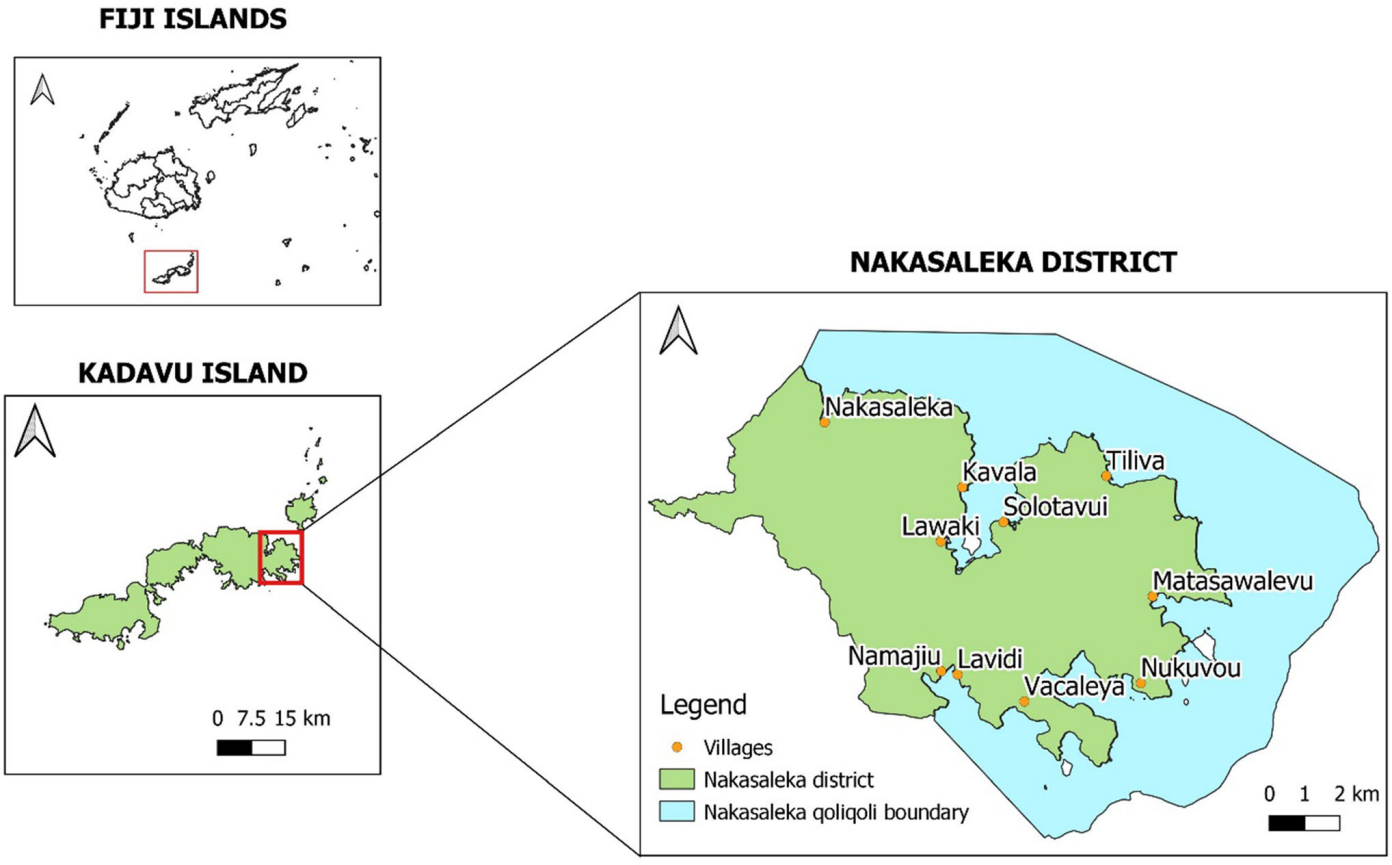
Nakasaleka district of Kadavu Island, Fiji

Nakasaleka is one of nine districts that make up the province of Kadavu (Fig. 1), a volcanic island arc located approximately 100 km south of Viti Levu, in the Fijian archipelago (Nunn 1999). The province comprised the third largest island in Fiji (Kadavu), and a scattering of islands to the north and east of it. The island group is bordered on the southern and eastern side by the Great Astrolabe Reef, one of the longest barrier reef systems in the world. Kadavu province has a land mass of 472 km^2^ and 31 registered customary fishing rights areas or *iqoliqoli,*^1^ covering an area of 719 km^2^. Its total population of 11,863 mainly reside in 75 villages and settlements (Kadavu Provincial Office, 2020 census, unpublished). Most economic activity is based on agriculture and fisheries, largely undertaken at subsistence level (Robertson et al. 2020). The major cash crop for the province is *yaqona* (kava, *Piper methysticum*), from the roots of which a ceremonial drink is made, also popularly consumed as part of various types of social gatherings (Sofer 2007 in Robertson et al. 2020), and which is sold (and sometimes exported) in its dried or powdered forms.

**Fig. 2:**
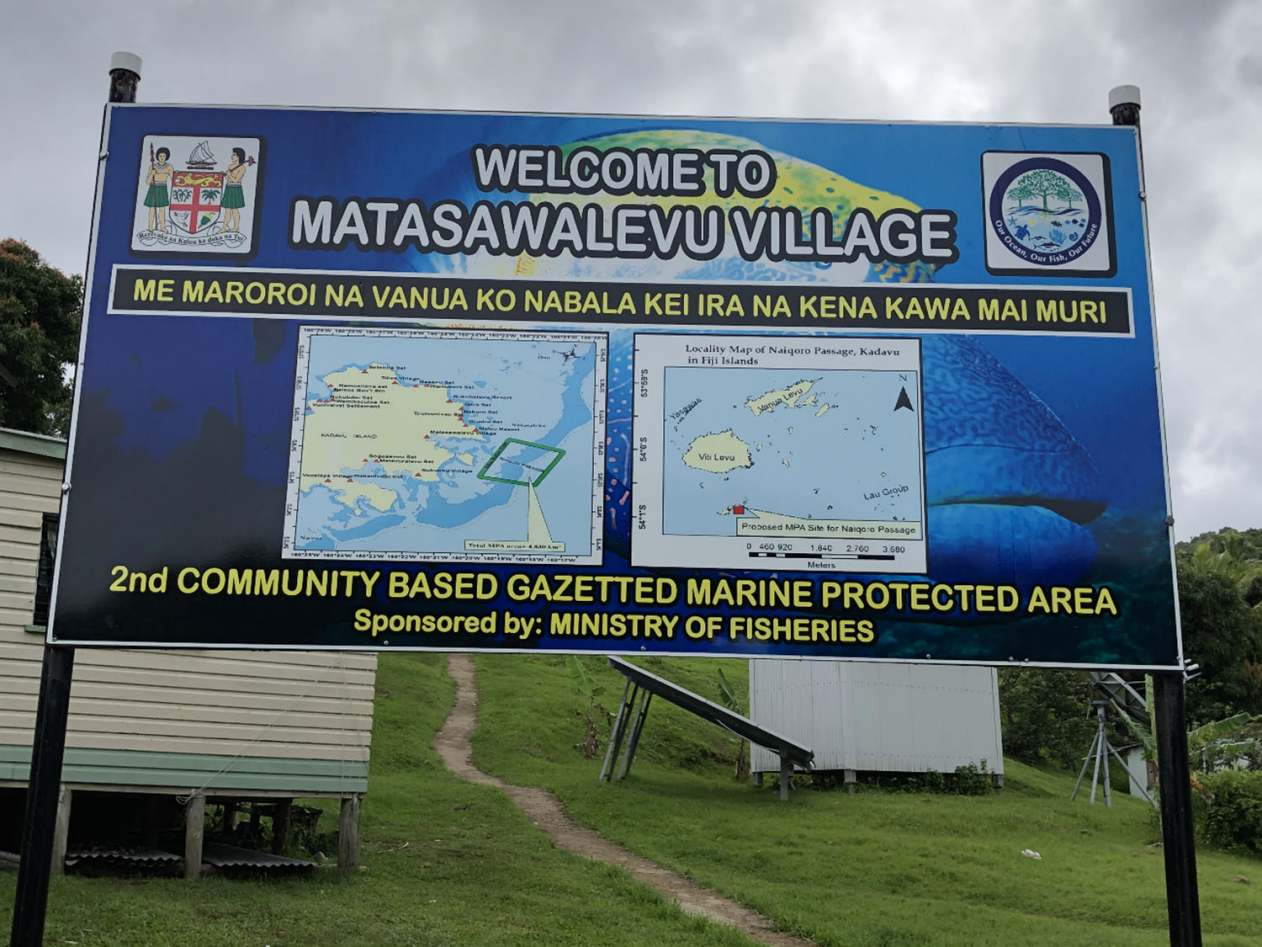
Information boards for the gazetted MPA at the entrance of Matasawalevu village (2019). ©Elodie Fache / IRD

Nakasaleka district at the eastern end of Kadavu island contains 19 villages and a number of smaller settlements, with a total population of 1524 in 2020 (Kadavu Provincial Office, unpublished data). The population in the district is predominantly *iTaukei* (Indigenous Fijian) in all villages and settlements. Inter-island ferry services from Kavala Bay provide a regular direct link to Suva, the national capital and a growing urban area containing almost a third of the country’s population. In this district’s waters, off Matasawalevu village, the Naiqoro Passage Spawning Aggregation Marine Reserve (4.83 km^2^) was officially launched in 2018 by the Fijian Government, with the purpose of “conserving, protecting and maintaining the biodiversity and productivity of the species of fish, sharks, rays, cetaceans, sea turtles and all marine organisms including coral and holothurian species within the demarcated area”^2^ (Fig. 2). This reserve is locally called a ‘gazetted MPA [Marine Protected Area],’ as its creation has been by way of specific regulations (*Fisheries (Naiqoro Passage Spawning Aggregation Marine Reserve) Regulations 2018*), brought into force by being published in Fiji’s Government Gazette.^3^

### Structured interviews: Household survey of marine resource catch and monetary value

A face-to-face household survey was conducted in October and November 2019 to estimate the amount and value of marine resources harvested by coastal communities in Nakasaleka district. The structured interviews aimed to investigate local fishing and gleaning practices and harvests. The main objective of this survey was to derive estimates of the annual catch and the monetary value of all resources harvested (finfish and invertebrates) by coastal communities in Nakasaleka district, for both commercial and subsistence fishing or gleaning.

The questionnaire (Appendix S1) was partly based on an earlier study conducted in south-west Madagascar (Barnes-Mauthe et al. 2013) and can be applicable to other data-poor fisheries in the western Pacific region. The survey collected information on the:

- number of fishing or gleaning days over a two-week period;
- types, amounts, and fate of marine resources harvested, and;
- how the catch changed according to the weather (perceived by the fisher or gleaner as ‘good’ or ‘bad’) and season (cyclone vs non-cyclone) based on experience over a number of years (see Appendix S2-A).

Marine resource users were categorized as fishers, fisher-gleaners, or gleaners only. Fifty adult respondents for each category were sought as this was shown in previous studies to provide a balance between efficient use of survey effort and maximal accuracy of estimates obtained (Barnes-Mauthe et al. 2013). These authors found that further sampling after fifty samples began to offer diminishing returns regardless of the variability of the surveyed variable. Although the population size in Nakasaleka district was smaller than that of the Velondriake study site in south-west Madagascar^4^, we aimed for a sample size of 50 respondents per category to ensure a highly robust sample of each type of marine resource user was collected in this study. Respondents were selected randomly within each village of the district (*n* = 19). Interviews were conducted either at the household or in the village hall.

Interviewees were asked to provide estimates of their catch for a range of finfish and invertebrate categories, and information about respective habitats (Appendix S2-B). Fishers were asked about three types of fishing methods: hook and line fishing, gillnet fishing, and freediving with a speargun. Speargun fishers who regularly sold their catch outside of the village were predominantly fishing at night with waterproof torches. However, the differentiation between day-time and night-time spearfishing was not recorded during the survey. The use of resources harvested by both fishers and gleaners was recorded as either sold, shared, or kept for food. For catch that was sold, fishers were also asked if the sale was to a vendor (e.g., a middleperson or a market seller) or to locals, and whether the catch was sold fresh, dried, or salted.

Although one person per household was interviewed, the gender and main sources of income of other adults (aged 16 or over) in the household were recorded as well as if they were fishing and/or gleaning. This enabled the calculation of the mean number of fishers, fisher-gleaners, and gleaners per household for the district using data from 19 villages. Adults recorded per household were those who cooked and ate together. Differences in fishing levels between villages, either due to the amount of resources harvested per person or the number of people fishing or gleaning per village, were not investigated in this study.

The mean catches of each finfish and invertebrate category were calculated for 156 households, then extrapolated to represent the total amount of marine resources harvested for the population of Nakasaleka district.^5^ Further information on the catch and monetary value calculations are provided in Appendix S2-C.

In addition to fishing or gleaning catches, information on livelihoods, sources of income, the consumption of fish and other types of protein, and ‘totem fish’^6^ were collected from interviewees. This study integrates only part of this information, specifically that on ‘totem fish’ and the sharing of marine resources. In terms of responses by women on ‘totem fish,’ this study did not separate between their own totem (the one they were born with) and that of their husband’s family/clan/village (in which they currently live), which should be examined in future studies.

### Semi-structured interviews: Sociocultural values^7^ of coastal fisheries

During the same time period, ten semi-structured interviews (7 recorded^8^ and 3 unrecorded) were conducted in three villages of Nakasaleka district: six in Matasawalevu, one in Lagalevu, and three in Kavala. The interviews addressed various topics related to fisheries and their management, including socioculturally significant marine fauna, changes in fishing practices over time, local perceptions of the (ecological and social) impacts of the Naiqoro Passage Spawning Aggregation Marine Reserve, and the (unpaid and unrewarded) role of local honorary fish-wardens in monitoring the marine reserve (see Box 1). They were conducted in the *iTaukei* language, English, or a mix of both languages, and lasted in average about an hour. The selection of the interviewees aimed to include both men and women in the sample, from different age groups and backgrounds, but all with some (extensive or more limited) fishing experience in their lifetime, as well as at least one elected village leader, one religious leader, and one fish-warden (Table 1). The identification of people meeting these criteria was done upon our arrival on site, in discussion with provincial staff, and based on the interest and availability of villagers.

**Table 1:** Additional information on the semi-structured interviews

Category and notes	Number	Gender
Village Headman (***Turaga ni Koro***) who was also a fisher and a fish-warden, but is not counted as such in the table below	1	Male
Methodist Pastor (***Talatala***) then in his mid-thirties, originally from Bua province, but based in Nakasaleka district for four years at the time of the interview, and going fishing only from time to time (“I’m from Bua, this is the place where I know how to fish,” i.e., not here in Kadavu, he told us)	1	Male
Fish-warden who was also a fisher, but is not counted as such below in the table	1	Male
Fishers (roughly 50 years old)	2	Male
Fishers (roughly 30 years old)	2	Female
Elderly people who used to fish a lot, but now rarely do so due to their age	3	1 Male, 2 Female

All recorded interviews were fully transcribed, with the parts conducted in the *iTaukei* language firstly translated into English. When the interviews were not recorded, notes were taken during the encounter and completed immediately afterward

For this paper, we have extracted from these interviews the data highlighting and contextualizing the sociocultural significance of specific taxa, relying on previous research experiences in Fiji and a triangulation with secondary sources. These additional qualitative data were intended to open up reflection avenues on the non-economic values of coastal fisheries within Nakasaleka district, based on the perceptions and practices of specific individuals. One limitation to the study is the reduced number of semi-structured interviews we were able to conduct until November 2019, which—although very rich—do not allow for extrapolation of the results to district level.

For the purpose of this study, everyday fishing activities were at the center of our attention. Thus, the household survey as well as the semi-structured interviews did not ask or account for fishing for special occasions (e.g., weddings, funerals, Christmas), which are often substantial and outstanding (compared to daily activities) in terms of volume and kind of species caught. This can be seen as a limitation of the study design, especially considering the high sociocultural value of these events (and hence also of the food served during these), which should be overcome in the future by follow-up investigations.

Box 1 Fish-wardens Fiji’s *Fisheries Act 1941* states that “The Minister [for Fisheries] may appoint honorary fish-wardens whose duties shall be the prevention and detection of offenses under this Act and the enforcement of the provisions thereof” (https://www.laws.gov.fj/Acts/DisplayAct/628#). In 2020, fish-wardens were officially presented as having three key roles: preventing, detecting, and enforcing the provisions of the *Fisheries Act*, after being trained to do so by the Ministry of Fisheries (https://www.fiji.gov.fj/Media-Centre/News/Feature-Stories/Fish-Wardens-Learn-to-Maintain-Resources). Yet, scholars have described their role as follows: “undertake surveillance work within their customary areas on behalf of the owners of customary fishing areas [or *iqoliqoli*],” and described their involvement as illustrating “the commitment of coastal communities to the proper use of their customary fishing areas [or *iqoliqoli*]” (Veitayaki 2008, p. 124). In Matasawalevu, according to the interviewees, following the creation of the Naiqoro Passage Spawning Aggregation Marine Reserve by the Ministry of Fisheries, the latter came to conduct a workshop and appointed all the participants (about 25 men) as fish-wardens, who each received a ‘certificate of authority’ delivered by the Permanent Secretary for Fisheries. This number of fish-wardens is unusually high. Usually there are two per village, reflecting the value and relevance of the marine reserve on both a local and national level.

## Results

A total of 157 people were interviewed during the household survey (one person per household) and classified as fishers (53.2%), fisher-gleaners (38.5%), and gleaners (8.3%).^9^ Among these interviewees, 44.9% were men and 55.1% women.^10^

### Fishing and/or gleaning

Fishing with a hand line was the most used method (44.1% of all fishers^11^), followed by freediving with a speargun (21%), and gillnet fishing (6.3%). Most fishers (71.3%) used one method, but almost a quarter of those interviewed used two methods (23.8%), and a few used all three (4.9%). The most common combinations were line and gillnet fishing (16.8% of all fishers), followed by freediving and line fishing (5.6%).

Most fishers who solely practiced freediving (96.7%) or who combined freediving and line fishing (87.5%) were men. Those solely using hand lines (68.3%) or gillnets (77.8%), as well as those using both lines and gillnets (91.7%), were predominantly women. The majority of fisher-gleaners were women (85%), while almost all interviewees solely gleaning were women (92.3%). For all those who practiced some sort of fishing (fishers only and fisher-gleaners combined), just over half (51.8%) were women.

### Catch, sale, and sharing

The total annual harvest of marine resources for Nakasaleka district, consisting of six finfish and nine invertebrate categories, was estimated as 2524 metric tonnes (Table 2), equivalent to 28.73 tonnes per km^2^ for the Nakasaleka *iqoliqoli*. This was mainly comprised reef fish (74% by weight), tuna (5.8%), coastal pelagic fish (4.5%), and crabs (4.4%). Mixed molluscan shells, sea urchins, and small pelagic fish are also important food resources.

**Table 2:** Estimated annual catch and monetary value of marine resources harvested in Nakasaleka District, Kadavu Province, Fiji

Resource category	# Surveys (n)	% Surveys	Total catch (m.t.)	% Weight	% Sold	Commercial catch (m.t)	Harvest revenue (10^6^ FJD)	% Harvest revenue	Total value (10^6^ FJD)	% Total value
Reef Fish	142	91.03	1856.67	73.56	54.49	1011.74	6.323	86.16	11.604	69.83
Small Pelagic	20	12.82	76.83	3.04	13.47	10.35	0.065	0.88	0.480	2.89
Coastal Pelagic	45	28.85	112.98	4.48	12.06	13.62	0.085	1.16	0.706	4.25
Tuna	13	8.33	146.32	5.80	68.30	99.94	0.600	8.17	0.878	5.28
Offshore Pelagic	9	5.77	19.38	0.77	28.17	5.46	0.033	0.45	0.116	0.70
Sharks and Rays	5	3.21	4.44	0.18	0	0	0	0	0.027	0.16
Crab	31	19.87	111.22	4.41	2.29	2.55	0.051	0.70	2.236	13.46
Molluscan shells	44	28.21	82.15	3.26	0	0	0	0	0.164	0.99
Sea Urchin	22	14.10	66.91	2.65	0	0	0	0	0.056	0.34
Trochus	8	5.13	12.54	0.50	61.49	7.71	0.053	0.73	0.087	0.52
Giant Clam	13	8.33	20.31	0.81	0	0	0	0	0.041	0.24
Lobster	11	7.05	6.26	0.25	80.18	5.02	0.105	1.44	0.132	0.79
Reef Octopus	12	7.69	5.22	0.21	0	0	0	0	0.063	0.38
Sea Cucumber	2	1.28	2.64	0.11	0	0	0	0	0.026	0.16
Reef Squid	1	0.06	0.12	0.01	0	0	0	0	0.002	0.02
Total finfish			2216.62	87.82	51.48	1141.11	7.11	97.13	13.81	83.11
Total Invertebrates			307.37	12.18	4.97	15.28	0.21	2.87	2.81	16.89
Total:			2523.99	100	45.82	1156.39	7.316	100	16.62	100

For the reef fish category, annual catch and monetary value were calculated using the response from fishers for their daily total catch of reef fish rather than the sum of each reef fish category

*# Surveys* Number of surveys, *m.t.* metric tonnes, *harvest revenue* value of sold marine resources, *10*^*6*^* FJD* 1 million Fijian Dollars

Almost half of the reef fish catch (46.34%) was harvested by spearfishing. On average, almost two-thirds (64.14%) of the reef fish harvested by semi-commercial fishers were sold (*n* = 48; SE = 0.036), although the proportion sold by individual fishers ranged from 25 to 100%. In fact, more than half (54.2%) of the semi-commercial fishers interviewed, who provided detailed catch data, were selling between 70 and 100% of their reef fish catch, with 12.5% selling all their catch. Just over half (55%) of all the reef fish harvested were sold locally.

Other commercially important resources were tuna (68% sold) and other offshore pelagic fish (28% sold). A high proportion of the lobster (80%) and trochus shell (62%) harvests were sold, although total catches for these were quite low (< 1% of the total catch weight combined). Only 2% of the crabs harvested were sold. Many invertebrates were not reported as sold, including sea urchin, octopus, squid, giant clam, sea cucumber, and mixed molluscan shells.^12^

Overall, 61.45% of the fishers, and 35.7% of the combined fishers and fisher-gleaners were selling a part of their daily catch. Of these semi-commercial fishers, 72.55% were men. Semi-commercial fisherwomen also represented 60.87% of all women fishing only (not gleaning), but only 18.92% of women who practiced both fishing and gleaning. Almost half (49.02%) of all semi-commercial fishers were freedivers using spearguns.

An assessment of the sharing of catch within coastal communities revealed that 53.85% of all fishers shared their daily catch with, on average, 20.20% of finfish catch shared.^13^ Fishermen shared less catch (15.42%) than fisherwomen (24.53%), with a lower proportion of men (48.53%) sharing than women (58.67%). Substantially more subsistence fishers (67.39%) shared their catch than semi-commercial fishers (29.63%), with the proportion of catch shared also greater for those not selling catch; 27.32% shared compared to 7.82% for those selling. The decision to share also depended on the daily catch for some fishers, who only shared if they had made what they deemed a ‘good’ catch.

Although the number of ‘gleaners only’ interviewed was low (13), the data available suggest that harvests of invertebrates were shared more often and in greater amounts than those of finfish. Most gleaners (between 66 and 75%) who were collecting mangrove crabs, sea urchins, and molluscan shells shared, on average, between 31 and 35% of the harvest for these invertebrates, in the community.

### Reef fish families

Mean catch proportions of reef fish by family are reported in Table 3 for catches by individual fishers. Mean values do not add up to 100% as fishers do not all catch the same fish families. Figures highlighted in bold in Table 3 represent the five reef fish families with the highest proportion of the daily catch, and the most often reported being caught (in terms of the number of responses) for the three types of fishing and for all types combined. Acanthurids (surgeon and unicornfish) and Lethrinids (emperors) were consistently important components of daily catch for all fishing techniques. Unicornfish (*Naso unicornis*) made up a substantial proportion of the catch for speargun fishers when recorded (mean = 30. 1%; *N* = 10; SE = 3.71). Scarids (parrotfish) and Serranids (groupers) also made up a high proportion of the catch for speargun fishers, while Lutjanids (snappers) and Serranids were a substantial proportion of catches using handlines. The ‘other’ category for four reef fish families combined often made up a high proportion of the daily catch for all fishing techniques apart from speargun fishing. These were primarily Holocentrids (soldier or squirrelfish), Leiognathids (ponyfish), Terapontids (grunters), and Gerreidae (silver biddy). The highest proportions were recorded for Acanthurids (49.25%) and Mugilids (mullets), with 43.77% for fishing with a handline and gillnet (albeit both based on small sample size, *n* = 2).

**Table 3:** Catch proportions for selected reef fish families expressed as mean percentage of total daily catch weight per individual fisher for three specific types of fishing and for all types combined

Reef Fish Family	Trophic Group	All Fishing^a^			Freediving / Speargun			Handline			Handline and Gillnet		
		Mean %	*N*	SE	Mean %	*N*	SE	Mean %	*N*	SE	Mean %	*N*	SE
**Scaridae**	Herbivore	**29.32**	**58**	1.75	**28.31**	**22**	2.51	n.d	n.d	n.d	19.00	4	4.21
**Lethrinidae**	Invertivore	**32.11**	**102**	1.93	**25.94**	**15**	4.23	**39.51**	**32**	2.91	**30.74**	**15**	4.22
**Acanthuridae**^**b**^	Herbivore	**35.64**	**46**	2.74	**36.32**	**19**	5.03	**28.84**	**9**	3.75	**49.25**	2	0.75
**Serranidae**	Piscivore	27.36	**83**	1.71	**24.01**	**15**	3.98	**27.06**	**26**	2.23	22.53	**11**	5.10
**Siganidae**	Herbivore	16.40	19	2.93	**18.79**	**9**	4.27	n.d	0	n.a	**22.67**	4	6.32
**Lutjanidae**	Piscivore / Invertivore	22.48	**44**	2.89	5.43	3	1.22	**24.74**	**16**	5.03	21.20	**8**	5.34
**Haemulidae**	Invertivore	9.52	9	1.44	5.49	3	1.88	12.50	1	n.a	16.67	1	n.a
**Labridae**	Invertivore	9.88	15	1.49	9.61	2	7.07	9.68	6	2.62	10.51	4	2.68
**Balisitidae**	Invertivore	16.38	35	1.60	7.74	3	1.77	19.10	13	3.21	10.81	5	1.61
**Mullidae**	Invertivore	13.38	30	1.25	13.27	4	3.49	10.18	3	3.44	18.45	**9**	2.47
**Mugilidae**	Detritivore	**36.80**	7	7.52	n.d	0	n.a	n.d	0	n.a	**43.77**	2	0.67
**Other**^**c**^	Multiple	**32.41**	24	4.39	14.88	2	8.21	**28.81**	**9**	6.13	**27.46**	**8**	7.51

*N* number of surveys, *SE* Standard Error, *n.d.* no data, *n.a.* not applicable

^a^The ‘All Fishing’ category includes the three categories provided in the table plus the following: gillnet; freediving/speargun and handline; freediving/speargun, handline and gillnet

^b^Includes Unicornfish (*Naso unicornis*) targeted by speargun fishers

^c^Other fish families caught were Holocentridae, Leiognathidae, Terapontidae, and Gerreidae

The main findings for reef fish catch proportions support the general assumptions of gear selectivity for trophic groups for the main types of fishing gear used. Handline fishing catches contain a higher proportion of piscivores and invertivores, while spearfishing catches depend on the preference of the fisher for fish that is either sold (higher price) or retained for consumption (preferred taste). It was not possible to discern the selectivity of gillnets from the data available, but it was noted that gillnets were generally deployed in nearshore areas such as mangroves where fish including mullets, goatfish, and grunters or silver biddy were often caught.

Table 3 also indicates that all those fishing were not generally targeting particular families, but harvest reef fish from a range of families and trophic groups. This was supported by responses to a specific question in the household survey (Appendix S1, p. 5), which found that 91.3% of fishers were not targeting particular types of reef fish (*n* = 115).

### Other finfish categories and invertebrates

Although reef fish made up most of the finfish catch in Nakasaleka district, other categories of finfish also provided an important part of the catch, often on a seasonal or sporadic basis. Coastal pelagic fish comprised 5.1% of the total finfish catch by weight (Table 2), and were commonly harvested, especially trevallies (Carangidae), but also needlefish (or long tom, Belonidae) and barracuda (Sphyraenidae). Small pelagic fish such as halfbeaks (Hemiramphidae) were often caught by women using a handline, while others such as little priest / baelama anchovy (Gerreidae) provided a seasonal harvest, usually between January and March, and were sometimes used as live bait when trolling for pelagic fish.^14^ Another seasonal catch is tuna, mainly yellowfin (*Thunnus albacares*), targeted primarily commercially between October and March.

A wide selection of invertebrate taxa was harvested, mainly by gleaning (predominantly by women), and provided a significant source of food for the villagers (Table 2). Mangrove crabs were particularly important in Nakasaleka district, with the vast majority retained for local consumption.

### Monetary value

The total monetary value of the marine resources was estimated for both those sold commercially (harvest revenue) and those not sold (consumed by the fisher’s household or shared). The monetary value of sold resources was based on the price at first point of sale by fishers, either per bundle or per kg. The monetary value of retained resources was estimated from local ‘farm sale’ prices for each resource category (Table 3), provided by key local informants.^15^ Further information on fish values is provided in Appendix S2.

The total annual gross value of marine resources harvested (Table 2) was estimated to be $16.62 million Fijian Dollars (FJD), equivalent to $7.61 million USD for a currency conversion dated October–November 2019. Reef fish were the most valuable resource, making up 86% of the harvest revenue ($6.32 million FJD), and 70% of total value ($11.6 million FJD). Tuna was the second most commercially valuable marine resource ($0.6 million FJD) and made up 8.2% of the harvest revenue. In terms of total value, crabs were the second most valuable resource ($2.2 million FJD; 13.46%), but were less than 1% of the harvest revenue. The only other categories to exceed 1% of the total value were tuna (5.28%), coastal pelagic fish (4.25%), and small pelagic fish (2.89%). Overall, finfish made up most of the commercial harvest with 97.13% of the harvest revenue. Although less than 5% of all invertebrates were sold, they did comprise 16.89% of the total value of marine resources harvested.

### Non-economic significance of finfish and other marine fauna

Out of the 157 individuals interviewed as part of the household survey, 114 gave information about ‘their totem fish’ (Table 4), with this category referring to finfish, invertebrates or other marine fauna (such as sea turtles). Five ‘totem fish’ were mentioned more than five times: *saqa* (trevally, *Caranx* spp.), *matu* (silver biddy, *Gerres* spp.), *tunadi* (unidentified small mangrove fish), *vaya* (e.g., little priest, *Thrissina baelama*), and *qari* (mangrove or mud crab, *Scylla serrata*), which together made up 72% of the responses. *Saqa* was by far the most recorded ‘totem fish’ (36% of all responses). The ‘other’ category consisted of a further 22 different ‘totem fish,’ with all but one (*ura*—shrimp: *n* = 4) given as one or two responses. Those with two responses included reef fish, such as *ta* (unicornfish, *Naso unicornis*) and *kabatia* (black-spot emperor, *Lethrinus* spp*.*), as well as *seasea* (an annelid worm found in soft sediment), *vonu* (sea turtle), and *qio* (shark).

**Table 4:** The most commonly recorded ‘totem fish’ in Nakasaleka district

Fish totem		Number	Percentage
*iTaukei* name	Common name		
Saqa	Jack/Trevally	41	35.96
Matu	Silver biddy	15	13.16
Tunadi	Not known	11	9.65
Vaya	Little priest	8	7.02
Qari	Mangrove crab	7	6.14
Others^a^		32	28.07

^a^Taxa mentioned less than five times

The semi-structured interviews confirmed the local sociocultural significance of two of these ‘totem fish,’ namely *saqa* and *vaya*, while providing local fishing knowledge including information about the fishing techniques and fishers’ perception of the status of these finfish (see Box 2).

Box 2 Importance of not’missing the boat’ (Johannes et al. 2000) Fishers’ knowledge and perceptions on the state of fisheries provide both quantitative and qualitative information that expands the available scientific data, helping to enable the implementation of strategies to sustain fisheries and conserve ecosystems (Mclean et al. 2022). The wealth of local ecological knowledge (LEK) that fishers possess—including of Indigenous fishing knowledge (IFK), rich but rapidly eroding in Fiji (Kitolelei et al. 2021)—is indeed a powerful tool to help understand coastal fishing communities as socio-ecological systems (Salpeteur et al. 2017), complement scientific research (Turner et al. 2015), and inform coastal governance and management (Barclay et al. 2017; Sjostrom et al. 2021). In this way, LEK transmission to younger and future generations could also be ensured (Kitolelei et al. 2021). The reliability of qualitative LEK information has been questioned by fisheries scientists (e.g., Soto 2006), although prejudices against fishers’ knowledge are also thought to be embedded within the structures of mainstream fisheries science (Hind 2014). Yet, ignoring fishers’ knowledge can lead marine researchers and managers to put fishery resources at risk or to unnecessarily compromise the welfare of resource users (Johannes et al. 2000). Comparison of both quantitative and qualitative fishers’ knowledge with more systematic quantitative fisheries data collection could be an approach to assess the reliability of the qualitative information collected, while also assessing the local meaningfulness of the quantitative fisheries data.

### Saqa

When asked about fish that are abundant at a certain time of the year, an 80-year-old woman who lived in Matasawalevu most of her life mentioned *saqa*:

The *saqa* comes through the reef passage and into the *jiro* [similar to a pond but seawater can flow freely to and from this pond – as explained by the interviewee] and when it drinks or swims in freshwater it becomes slow and it’s easy to spear it. So, people go and catch the *saqa* in the *jiro*. […] Before when my husband was alive, he used to go spearfishing and bring our *saqa*.

Yet one of Matasawalevu’s fish-wardens thought that the abundance of “big *saqa*” had greatly declined over time in the village’s waters, but that an increase was now being observed due to the establishment of the Naiqoro Passage Spawning Aggregation Marine Reserve:

Even the fish we no longer used to see they are coming again like the big *saqa*. At some point they were gone, but ever since the creation of the Naiqoro Passage reserve those big fishes are coming back. That’s one of the benefits of the gazetted MPA.

He then mentioned that the ‘totem fish’ of his clan (*yavusa*) is *saqa* (a status that is not accompanied by a customary ban on eating this fish), which might explain his heightened awareness of this fish. In Kavala, the husband of an elderly interviewee participated in part of the conversation, and formulated his own connection to this fish in the following way:

See, all these people, they have their own fish. Traditional. When you are [*iTaukei*] Fijian, they can tell you that […]. *Saqa,* my fish. *Ika va turaga* (chiefly fish), that is for the chief.

Another dimension of the local significance of *saqa* was expressed by a passionate master fisherman^16^ in his fifties, living in Lagalevu. He knew the behavior of his targeted species well and presented his fishing activities as a kind of encounter between his knowledge as a fisherman and that of the fish:

Some species do get tired quickly, yeah, in some species, it will take a while, trevally it will take a while to tire […]; the other ones they just stay on the surface eh, and trevally, that’s a problem as they go straight down, yellowfin and trevally, straight down […]. [When trolling,] letting the line out, and tracking behind the boat […], depending what fish, you have to know it; sometimes it’s too slow, the fish is smart, […] I think they have gone to University, got a University degree…

This last humorous remark illustrates that, in Nakasaleka district, fish are not only seen as ‘economic resources’ but as smart counterparts in the everyday struggle for food and income, hence a human–fish relationship that acknowledges and respects the same struggle on both ends of the line.

### Vaya

The above-mentioned elderly woman described herself as “one of the women who is very smart in net fishing” (in general, not specifically gillnet), even though she no longer fished because of her age. When asked to share some stories about any fish having a specific meaning for her, she spontaneously mentioned *vaya*, caught by women only along the shore, using a fishing net:

They come in schools and that’s when we go and catch them using the *taraki* [a type of fishing net] and scoop them from the water. […] There will be plenty of us [women] and we scoop the fish using our *taraki*. […] For *taraki* I have my own, another has her own, another has her own, there’s plenty and when one has the fish jumping in the net we run to put our *taraki* together to hold the fish. When the fish want to escape it’s too late. […] Only the women. Along the shore just out here and when the tide is coming in the bigger fishes [such as *saqa*, as she later explained] come and they eat the *vaya*. […] Now, sometimes people bring me *vaya* and I tell them yes, only the *vaya* is nice. They always bring me a basin of *vaya* and I thank them because I like eating it. It is a small fish but it tastes good. […] When someone goes fishing and sees the *vaya*, they come back and tell us and we all go. We get our *taraki* and go. I just watch them from here I don’t go fishing for *vaya* anymore. […] Everyone who went to catch *vaya* they give it to their families because there is plenty. I always thank them for bringing mine. Every household will eat *vaya*.

This fish is important to her (and to the women who catch it) as a tasty and highly valued food. It also has a special significance in Matasawalevu: she explained that “the *vaya* is the *ika va turaga* (chiefly fish, i.e., fish that fishers are expected to present/give to the chief) in this village and the *ika ni masi* (‘totem fish’ of the village, which can be a seasonal fish used for traditional functions, such as when a chief is installed) is the *vaya* too” (see also Gordon 2013). This status might be related to this woman’s emphasis on sharing: sharing information about the presence of this fish, sharing the fishing effort (based on collaboration between women), and sharing of the catch.

Another elderly woman, interviewed in Kavala village, also mentioned *vaya* as a significant fish for her (“that’s my fish”); a fish that “is nursed” in the mangroves “so it can grow big enough” and be caught; a fish that was largely shared among villagers, who consumed it boiled, fried, or cooked with coconut milk.

## Discussion

The monetary value of marine resources harvested by coastal fishers and gleaners estimated by this study is higher than previous estimates in Fiji. Our estimate of gross value was $16.6 million FJD for one district of Kadavu in 2019. If we tentatively extrapolate our estimates to cover Kadavu province (nine districts), based on household numbers, the gross value for inshore fisheries rises to $125 million FJD for the province. This means that the monetary value estimated by this study for Kadavu province is almost the same as the national estimate by the MACBIO project (gross value of inshore fisheries, subsistence and commercial combined) for Fiji’s 14 provinces ($113 million in 2014 equivalent to $130 million in 2019 accounting for annual inflation^17^) (Gonzalez et al. 2015). If we extrapolated our estimate to the national level, it would be substantially higher than the MACBIO estimate. The study conducted in Madagascar also derived monetary value estimates that were considerably higher than existing valuations at the time for Madagascan coastal fisheries (Barnes-Mauthe et al. 2013).

The MACBIO study was mainly based on the estimates made by Starkhouse (2009) for subsistence and commercial coastal fisheries. This study involved extrapolating the findings in 12 villages to provide a national estimate, based on economic models derived for both artisanal and subsistence fisheries, as well as using a number of sources of secondary information. Some important inshore marine resources were also excluded from the artisanal fishery analysis including mangrove crabs and inshore pelagic fish such as mackerel. The commercial inshore fishery value estimate by Starkhouse (2009) was regarded as under-representative of the total volume or value of the national artisanal commercial fishery (Gonzalez et al. 2015).

However, the extrapolation to the provincial level assumes that the level of marine resource harvesting and dependence is similar across all nine districts in Kadavu. The amount and composition of marine resources harvested in districts other than Nakasaleka is likely to vary depending on the size of the customary fishing rights areas (*iqoliqoli*). The Nakasaleka *iqoliqoli* is one of the largest in the Kadavu province (87.87 km^2^), with extensive areas of reef and lagoon within the Astrolabe barrier reef system (Fig. 1). However, as the extrapolation is based on the number of households in the province and the expected similar dependence on marine resources for the people of Kadavu, the size of the *iqoliqoli* is not likely to be the only factor when estimating marine resource catch and value at the provincial level. Productivity will also depend on the health of the inshore marine ecosystems, demographic data and dynamics, the effort put in by marine resource harvesters (which might be linked, for instance, to the accessibility of fish markets and the effort devoted to farming activities), and the presence (or absence) of fish aggregating devices (FADs), which can increase the availability of pelagic finfish. The spatial area of mangroves and seagrass beds within an *iqoliqoli* will also affect the proportions of marine resources harvested, especially for some invertebrate categories such as mangrove crabs.

Nakasaleka district also contains the Naiqoro Passage Spawning Aggregation Marine Reserve, which is a no-take ‘gazetted MPA,’ well respected by local fishers but not strongly enforced to prevent potential poaching by outsiders. This marine closure (4.83 km^2^) is not thought to have affected the catches of local fishers since its implementation in 2018. If well managed, it is likely that reef fish populations for some exploited species will increase in this reserve over time, resulting in spillover to the surrounding area. This may enhance the catches of some local fishers, resulting in an increase in the value of marine resources harvested within the *iqoliqoli* of Nakasaleka district.

If we assume that all fishers and gleaners were only harvesting marine resources from the *iqoliqoli* of Nakasaleka district, it would be possible to calculate the catch of most marine resource categories per unit of spatial area. For example, the total annual reef fish catch would be equivalent to a harvest value of 211.32 kg ha^−1^ year.^18^ This assumption is likely to remain true for some categories such as invertebrates collected by gleaning but is less applicable for finfish categories such as reef fish and coastal or ocean pelagic fish. The main reason for this is that some fishers were fishing in adjacent *iqoliqoli* on occasion, especially in Ono district’s *iqoliqoli* as a mutual relationship exists between the communities of the two districts to access each other’s fishing grounds. Larger pelagic fish such as tuna or walu (spanish mackerel, *Scomberomorus commerson*) were also caught beyond the outer reef slope in open water, which does not generally fall within designated *iqoliqoli*.

The importance of reef fish for Nakasaleka district was clearly shown by this study, making up 74% of the catch by weight, 86% of the commercial value, and 70% of the total value. However, pelagic fish are also an important resource, especially seasonally for tuna and some small pelagics (14% of the catch, 11% of commercial value, and 13% of total value). Invertebrates provide an important food source at the subsistence level, particularly crabs, sea urchins, and molluscan shells, which can also be harvested when weather prevents or restricts fishing. Apart from lobster and trochus, the proportion of invertebrates sold was very low (0–3%) with most retained for local consumption. This suggests that there is potential to increase the proportion of some invertebrate catches (e.g., mangrove crabs, octopus, and sea urchins) that are sold if fishers or gleaners can readily access a market such as Suva.

The focus on reef fish as a key source of income and food in the district, and the prominence of spearfishing at night to catch a substantial portion (46%) of the total reef fish harvest, is of some concern. Spearfishing is regarded as an unsustainable fishing technique when unregulated (Gillett and Moy 2006). Fishing at night is likely to increase the level of unsustainability as reef fish are generally easier to catch, especially if fishers also target spawning aggregations. Although we did not ask fishers about the latter specifically, many did mention that a key time for night spearfishing was over the new moon, when some reef fish families are known to form spawning aggregations. One of the key reasons for officially protecting the Naiqoro reef channel near Matasawalevu was to prevent fishing on spawning aggregations of groupers (Sadovy 2011). Other management measures such as introducing size and catch limits have been suggested for spearfishing, which could work positively with the inherent selectivity of the method (Lindfield et al. 2014). Spearfishing at night was also recently banned in Lau province as part of the ‘Lau Resource Declaration’^19^ and is a potential option for Kadavu if supported by local communities.

Our estimates take into account the effect of season^20^ and weather^21^ on fishing and gleaning activities, which were both substantial. Inclement weather affected both the number of days and hours spent fishing or gleaning per day, the habitat fished, and the type of fishing practiced, thereby strongly influencing catches. Climate change effects on the incidence of inclement weather are likely to influence the amount of fishing or gleaning carried out by coastal communities on Kadavu. The fisheries sectors of Small Island Developing States (SIDS) have been identified as particularly vulnerable to climate change and climate variability (Monnereau et al. 2015), including in the tropical Pacific (Bell et al. 2011). The greater frequency or intensity of severe storms is a climate stressor. Future changes in the intensity, severity, and frequency of storms could threaten fisheries through disruption of fishing, damage to vessels and gear and, with other effects such as safety at sea, jeopardize the wellbeing of fishing households and coastal communities (Turner et al. 2020).

Our estimates do not take into account fishing for special occasions, catches of some local artisanal fishers who do not live in coastal communities, sport fishing by local tourism operations, commercial fishing by fishers based in Suva who travel to northern Kadavu (in particular to the waters of Ono or Nakasaleka districts),^22^ and longline vessels that fish close to the island.^23^ It would be necessary to follow up this work with a more comprehensive study that takes these additional fishing activities into consideration to improve the catch and value estimation for coastal waters of Kadavu.

Other limitations for this study in terms of the methods and extrapolation are likely. The catches for some of the marine resource categories were based on small sample sizes, increasing the likelihood of inaccurate values being used for the calculations. It is also likely that some fish or invertebrates may not have been recorded in the assessment, especially if they were seasonal and not reported by the interviewee at the time of the survey. Another example is the opportunistic collection of invertebrates for food by freedivers when spearfishing. Some types of invertebrates were recorded as collected by spearfishers such as lobster and giant clam but others such as trochus were likely missed. Variation in the detail recorded for fishing and gleaning between those conducting the interviews^24^ is also a possible limitation. Language may also have been a factor for one interviewer who was not fluent in the *iTaukei* language, although all marine resource categories and reef fish families were provided in both *iTaukei* and English on the survey form.

Although our catch estimates from household surveys were not validated by concurrent creel surveys, a previous study in Kadavu, in Ono district, concluded that reported estimates of the number and size of fish were sufficiently accurate with no significant difference between the recalled and observed (creel) contribution of the majority of finfish families and invertebrate taxa (Kuster et al. 2006). Catches and catch rates reported by households in Samoa also compared favorably with those observed in a concurrent creel survey (Hosch 2000). We are confident that the reported catches for Nakasaleka district are accurate for the broader marine resource categories recorded (e.g., total reef fish). However, over-estimation of the contribution of some reef fish families by a few of the respondents was observed in this study. Kuster et al. (2006) also found that the number of fish reported for the most abundant family (Lethrinidae) was over-estimated by respondents, with an underestimate for the numbers of fish caught more rarely.

Our household surveys also revealed the importance of women in terms of both fishing and gleaning, mainly to provide marine resources for local consumption in Nakasaleka district. For reef fish, women provided 22% of the annual catch, 40% of the subsistence catch, and 7% of the commercial catch. For gleaned invertebrates, women provided all of the catch for octopus, sea urchins, and mixed shells, none of which were sold. The household surveys thus contribute to filling the research gap on quantifying the contributions of women in the subsistence and commercial small-scale fisheries sector in Fiji, thereby helping to ensure that “women fishers and their contributions are visible, acknowledged and recognized” (Thomas et al. 2021, p. 8). On the one hand, our results do confirm the vital economic benefits provided by *iTaukei* women, particularly in terms of food security and income generation within their households, through the harvesting of marine resources (Kitolelei et al. 2021). On the other hand, like the household surveys carried out by Thomas et al. (2021), they show that in Kadavu province (not surveyed by these authors), women glean more (and so collect more invertebrates) than men, rarely practice freediving with a speargun, and sell less of their catch compared to men.

The sharing of harvested finfish and invertebrates is also an important aspect of village life, providing socio-ecological systems resilience (Dacks et al. 2020), and was a common practice for many fishers and most gleaners. The sharing of resources with relatives, other community members, and sometimes people from other groups (e.g., groups having common borders or ties) was, and to some extent remains, a pivotal *iTaukei* behavior and value (Nabobo-Baba 2006), ensuring that “the resources were efficiently used and that people looked after each other in times of need” (Veitayaki et al. 2014, p. 34). The sharing of the catch is of particular importance for socioculturally significant fish, such as *vaya*: it is both expected and much appreciated. In local views documented in other parts of Fiji, the sharing of (often seasonal) ‘fishes of value’ is even deemed necessary to ensure that these will remain available and abundant, whereas their selfish consumption or sale would result in their disappearance (Fache and Pauwels 2020). However, in Kadavu, the level of sharing for some resources such as reef fish may be decreasing as fishers sell more of their catch. We found that considerably fewer semi-commercial fishers shared their catch compared to subsistence fishers, with the proportion of the catch shared also lower for those selling. Higher market integration can result in weaker sharing networks (Dacks et al. 2020) and may also be linked to reducing resilience to food system shocks (Ferguson et al. 2022).

**Fig. 3:**
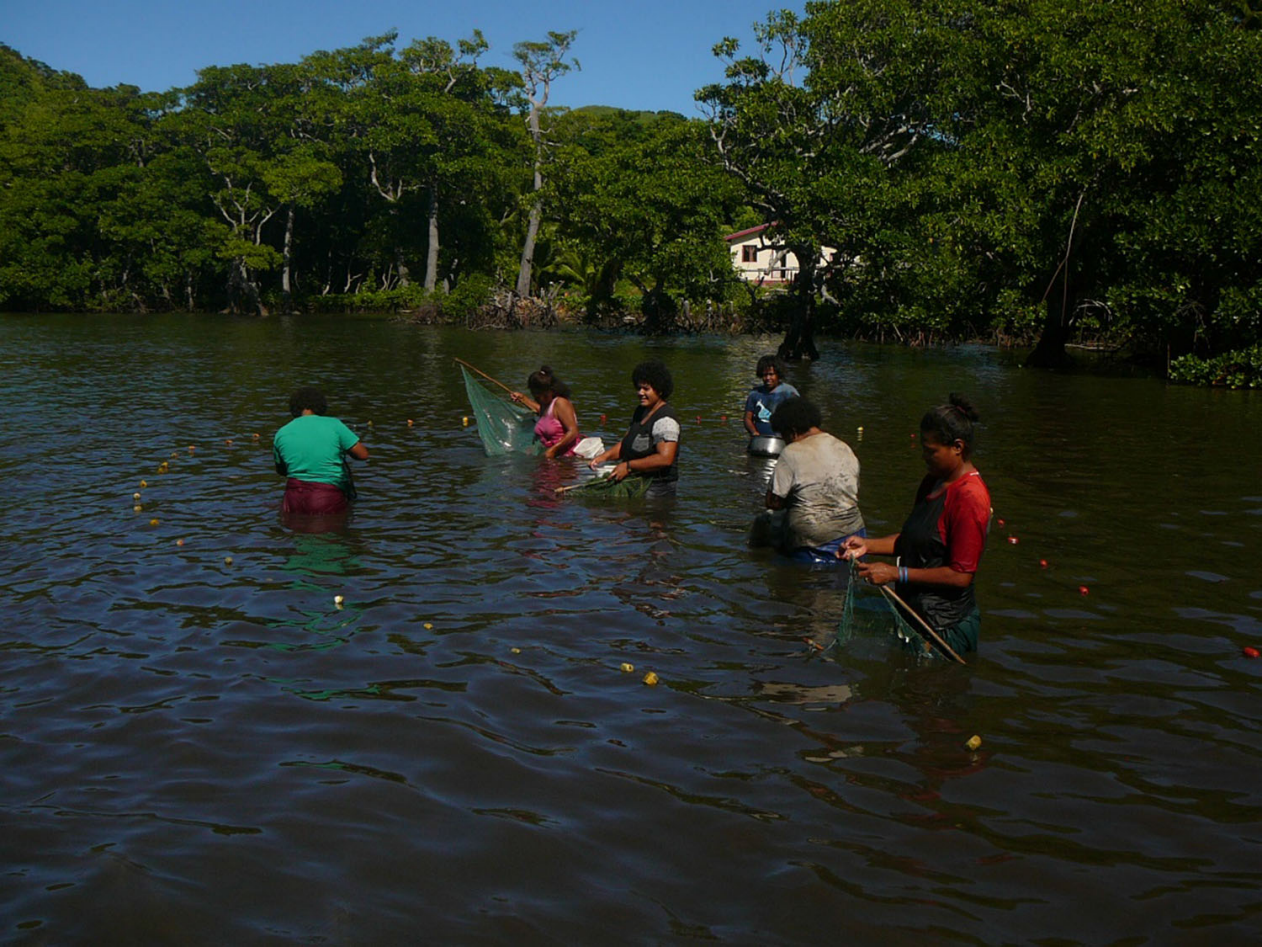
Women collectively fishing for *vaya* at Matasawalevu in May, 2019. ©Simon Harding / USP

While *vaya* is not sold commercially, in Matasawalevu, it is valued as “a readily accessible source of protein” (Gordon 2013, p. 338), and has other uses such as live bait for trolling. But above all, the inhabitants of this village (and potentially of other villages in Nakasaleka district), and especially the women, were very attached to it. We have been able to personally witness women’s collective catch of *vaya* in Matasawalevu (Fig. 3). As per Gordon (2013, p. 337), we have observed that “Women enjoy fishing [*vaya*] together; laughter and joking features prominently in this experience.” *Saqa*, on the other hand, is often sold by local fishers,^25^ while it is also regarded as a very smart marine being (or even a ‘social partner’ or counterpart rather than a ‘prey’?; Bataille-Benguigui 1988). Both finfishes have a high sociocultural significance, as a local delicacy, a ‘totem fish,’ and a ‘chiefly fish.’ They illustrate that the term ‘totem fish’ covers human–fish connections at the individual (“my fish”), clan (*yavusa*), and village levels, while being a key element of the possible relationships between chiefs and their people, seen (or even idealized) as reciprocal: “the people serve him [their chief], who in turn serves the people by redistributing the wealth while ensuring peace and abundance” (Pauwels 2015, p. 189). These ‘totem fish’ can be described as “symboliz[ing] *custodianship* and link between the community and the environment” (Ratuva 2007, p. 104), here coastal/marine areas. However, as they are targeted, caught, and locally eaten, they do not seem to be considered “sacred and representative of [people’s] ancestral being,” which would imply that “these fish are not eaten or disturbed because these are believed to invoke the wrath of the gods” (ibid., p. 94). Still, as important and highly valued ‘totem fish’, *vaya* and *saqa* can indeed be considered as ‘cultural keystone species’ (CKS; Garibaldi and Turner 2004; Noble et al. 2016; Kitolelei et al. 2021), and we argue for systematically taking into account these and other CKS in future value assessments of coastal fisheries. The recognition of such ‘totem fish’ as CKS could help these communities to maintain their cultural, social, and economic health and wellbeing, as well as the coastal ecosystem goods and services on which they depend, while motivating new, more equitable, and co-designed co-management strategies (Noble et al. 2016). This recommendation could be extended to other contexts, within and beyond PICTs, where connections between local and Indigenous communities and ‘totem fish’ or more generally ‘totem animals’ exist, such as in various African contexts (Mandillah and Ekosse 2018), for instance in Nigeria (Dagba et al. 2013), or in India (Singhal et al. 2021).

## Conclusion

This study has shown that estimating the catch and (both monetary and subsistence) value of marine resources in Fiji using household surveys is a useful approach that can provide reliable data at the district level, which can then feed into estimates at the provincial or national level. The approach is also applicable to coastal fisheries in other PICTs and could help to raise the profile of these fisheries for decision makers. Quantifying both sold and unsold harvested marine resources also highlighted women’s contributions to small-scale fisheries; increasing this recognition and their visibility remains critical to the design of inclusive and sustainable management approaches to coastal fisheries. In addition, the focus on unsold catches reveals that sharing of harvested fish and invertebrates, including of taxa of high sociocultural significance, is still an important activity for these coastal communities. The integration of dynamic sociocultural aspects is also essential for approaches that acknowledge local ways of valuing and make them more visible. However, the level of semi-commercial fishing and current spearfishing practices (especially at night) may contribute to the erosion of socio-ecological system resilience for coastal communities. Finally, we call for the recognition of ‘totem fish’ as CKS to improve future (co-)management strategies.

## Supplementary Information

Below is the link to the electronic supplementary material.

Supplementary file1 (PDF 1023 kb)

## Footnotes

1 The term *iqoliqoli* refers to the 411 demarcated and registered inshore water areas (approximately 30,000 km^2^ in total)—from the foreshore to slightly beyond the outer reef slope—over which customary fishing rights are held by iTaukei (Indigenous) Fijians. It reflects the national recognition of the iTaukei customary marine tenure system, which includes rights to access, use/harvest, and manage nearshore fishing grounds and all marine species within; the State, however, retains the ownership of the seafloor as the *iqoliqoli* fall within the territorial sea, as well as “the power to legislate or regulate resource use” therein (Sloan and Chand 2016, p. 77).

2 Fisheries (Naiqoro Passage Spawning Aggregation Marine Reserve), Sect. 9 of the Fisheries Act 1941, Legal Notice N°4, https://www.fiji.gov.fj/getattachment/b920c7d1-60e8-4b81-aae8-343b3d2c3730/LN-4---Fisheries-(Naiqoro-Passage-Spawning-Aggrega.aspx (Accessed on 26 July 2021).

3 See https://www.sas.com.fj/ocean-law-bulletins/fijis-minister-for-fisheries-has-created-two-new-marine-reserves-with-regulations-made-under-powers-conferred-by-section-9-of-the-fisheries-act-1941 (Accessed on 27 June 2022).

4 1524 people in Nakasaleka district, Kadavu, Fiji compared to 7500 people in Velondriake, south-west Madagascar.

5 Data for the number of households in the district were provided by the Kadavu Provincial Office.

6 A Fijian-English dictionary (Gatty 2009) explains that what iTaukei Fijians may call their fish, their plant or their bird (sometimes referred to as their *icavuti*) is often a taxon for which they have a high regard or which is associated with their ancestral spirit. In some cases, this taxon has some fertility significance, while in others, it is merely an iconic entity having an importance in local customs. Nakasaleka communities have been reported to use the phrase ‘totem fish’ to refer to such socioculturally significant fish (Gordon 2013).

7 This plural form, ‘values,’ is intentionally used, based on Le Meur et al. (2021) who highlight the tension between ‘value’ (singular form) and ‘values’ (plural form), i.e., between alienable/price and inalienable.

8 An audio recording of the interview was made using a digital recorder.

9 Although we did not achieve our target of 50 respondents for gleaners, we did interview a total of 73 people (gleaners and fisher-gleaners) who practiced some level of gleaning and had robust sample sizes (n > 10) for the majority of invertebrate categories. The inclusion of data with low sample sizes is useful for data-poor fisheries as it is an improvement to have even limited data as long as there is a clear understanding of the data’s limitations (Barnes-Mauthe et al. 2013).

10 One interview was not used in the analysis as the individual had stopped fishing in 2017.

11 The term ‘all fishers’ refers to both people solely fishing and those fishing and gleaning.

12 Multiple species of mainly intertidal gastropod, bivalve, and chiton molluscs.

13 Figures for the average amount of catch shared are calculated from all fishers or gleaners for that particular category, i.e., they include those who do not share their catch. For all fishers who do share, the proportion of catch shared was 37.51%.

14 Semi-structured interview with a village headman.

15 Key informants were residents (fishers and/or gleaners) of Matasawalevu village and the fisheries officer based at the Ministry of Fisheries station in Kavala Bay.

16 This fisherman explained to us: “I started my school in the city, in Suva. Then I did not like school so… […] So I have since then been fishing, I love fishing, I’ve sticked to fishing. […] I will fish till I am walking on a stick. […] Because I love the ocean, yeah. […] I don’t know when I’ll stop fishing, maybe if one of my arms is gone or something I don’t know but otherwise… […] If I’m still carrying myself around, I will still be in the ocean.”

17 Annual inflation rates sourced from the World Bank: https://data.worldbank.org/indicator/FP.CPI.TOTL.ZG?locations=FJ

18 The spatial area of the Nakasaleka *iqoliqoli* is 87.86 km^2^.

19 https://fijisun.com.fj/2021/06/10/lau-province-ban-night-spear-fishing-a-first-for-fiji/ (Accessed on 31 January 2022).

20 Fishers alter the gear used and the time spent fishing between the seasons. These types of variations in fishing and gleaning were not factored into the calculations of catch and value, and may also alter the totals derived for the district.

21 These two quotations from semi-structured interviews illustrate local perceptions of ‘good’ and ‘bad’ weather: “Good weather is when the sky is clear, the sun is out, there’s plenty stars in the sky at night. Bad weather if it’s raining, dark skies.” “For good [weather], good allows me to go outside, then it’s good, you know; when it’s bad [weather] then I can’t go out, so I just have to try fishing inside. […] Main thing is calm, yeah, doesn’t matter the rain, I carry my compass, just for the safety, it’s just the wind and the wave, I don’t like it…” The perception of ‘good’ or ‘bad’ weather by fishers or gleaners is likely to vary according to age, gender, whether the fishing is mainly commercial or for subsistence, and other factors (e.g., the person is from the district or from another area of Fiji). A better understanding of what the term ‘weather’ (draki) locally means/covers, and a comparison between fishers’ or gleaners’ perceptions of weather conditions (using a larger sample size) and meteorological data could determine the reliability and refine the interpretation of weather responses from resource users, and thus help to better define ‘good’ and bad’ weather conditions.

22 Interviews with Suva-based fishers for a separate study of value chains revealed that Kadavu is targeted by speargun fishers on some night trips (Harding, unpublished information).

23 Observed by one of the fishermen who was part of the semi-structured interviews.

24 Two of the interviewers were staff from the Kadavu Provincial Office, while the other three were from the University of the South Pacific.

25 40% of fishers who reported catching *saqa* were selling between 40 and 100% of their daily catch.
