# Supplementary material for: Marine resources and their value in Kadavu, Fiji
Source: Ambio. 2022 Oct 1;51(12):2414–30. doi: 10.1007/s13280-022-01794-0 (PMC9584011; doi:10.1007/s13280-022-01794-0)
Supplement: Supplementary file 1 — Supplementary file1 (PDF 1023 kb) [file 13280_2022_1794_MOESM1_ESM.pdf]

Ambio

Electronic Supplementary Material
This supplementary material has not been peer reviewed

Title: **Marine resources and their value in Kadavu, Fiji**

Authors: Simon Harding, Kalisiana Marama, Annette Breckwoldt, Ulamila Matairakula, Elodie Fache

Appendix S1. COMMUNITY-BASED HOUSEHOLD MARINE RESOURCE VALUE SURVEY IN THE NAKASALEKA DISTRICT, KADAVU

Goal: To better understand the value of marine resources harvested by coastal communities in Kadavu

Introduction to respondents:

- Introduce yourself and who you work for
- Explain the survey will take about 30 minutes
- State that information will be confidential:
- Will only release aggregated / anonymous data (no personal information)
- Final report will be available on the IMR website or can contact IMR or Kadavu Provincial office for a copy

Intro example:

Hi I'm [name] from the Institute of Marine Resources at the University of the South Pacific. We are undertaking an assessment of harvested marine resources for food and income in Kadavu to better understand their importance and value. Would you kindly assist us with this survey? It should take about 30 minutes. Thank you for your time.

Interviewer		Date of the interview	
Village name		Name of the data enterer	
Questionnaire number		Date of the data entry	

**People consent to the interview**	Name	Male / Female	Age	[1] Fishing only (Note if with net, free diving or with line) [2] Gleaning only; [3] Both
**YES** **NO**				

Do you have a fishing license? (Yes / No):____________________

NOTE : **FOR FISHERS ONLY – COMPLETE TABLES 1, 2 AND 4**

FOR GLEANERS ONLY– COMPLETE TABLES 3 AND 4

FOR FISHERS/GLEANERS – COMPLETE TABLES 1, 2, 3 AND 4

SECTION I: HOUSEHOLDS: Total number of fishers / gleaners

How many people are in your household? (Those who share a meals and cook together)

	Who are the people over 15 years old in your HH?	Male / Female	Does she/he both fish and glean?	Is her/his principal activity just fishing?	Is his/her principal activity just gleaning?
1					
2					
3					
4					
5					
6					
7					
8					

1. How many children under 15 years old go fishing or gleaning?

Income Sources

Note the number of the people in your HH who are involved in the activities in the table below and total weekly income for each activity

**Activities**	9. Number of people in the HH doing it	10. weekly income (or rank)
01. Net fishing, spear fishing, line fishing		
02. Gleaning		
03. aquaculture and sea farming / ranching		
05. selling food or drink		
06. farming		
07. selling handicrafts		
08. working in tourism		
09. working on resource conservation		
10. “doing business” = trading		
11. salaried employment		
12. marine transportation		
13. Other (specify)		
**Total amount of HH income**		

SECTION II FISHING AND GLEANING: Number of Fishing Days per Season

	Dry season (May – October)		Wet / Cyclone Season (Nov – April)	
	Good weather	Bad weather	Good weather	Bad weather
In each season, how many days are good days and how many days are bad days in one month (use week if month too long)				
**FOR THE FISHER**: in a good month for each season, how many days do you go out fishing? and during a bad month? (30)				
**FOR THE GLEANER**: in a good month for each season, how many days do you go out gleaning? and during a bad month?(30)				

Day for going out fishing / gleaning

Total no. of days for fishing in 2 weeks when:	Total number of days (=< 14 days)	Number of days for fishing only	Number of days for gleaning only	Number of days for fishing and gleaning
01 the weather is good				
02 the weather is bad				

For the fisher who said that they glean too. When you are gleaning?

	✓						
I glean during particular months / season		→	02 Number of the days to glean during a month in that season	Days		03 Which months / season?	Months
03 Every spring tide		→	04 Number of days gleaning over the spring tide	Days			
05 Sometimes on the spring tide		→	06 How many spring tides in a year (or 6 months)	Spring tides	→	07 Number of days gleaning over the spring tide	Days
08 Only if the weather is bad for fishing		→	09 When the weather is bad, I go gleaning:	[1] Every day	[2] once per 2 days	[3] once per 3 days	[4] once per week

Number of hours per day (24h)

How many hours do you spend when:	Hours spent fishing?	Hours spent gleaning?	Hours gleaning and fishing
The weather is good			
The weather is bad			

FISHER ONLY-TABLE 1. Fishing When you go fishing only (not gleaning), estimate all the catch for one day on a good (G), bad (B) or normal (N) day (**one row per habitat type - use extra pages)**

Main Fish type	Fishing site	Technique of fishing	Caught regularly or occasionally?	How many kg (or bundles) is your maximum, minimum and normal daily catch for each main fish type?			In the total (normal) catch, what proportion (%) or how many kilos or bundles are for …					For the catch you sell, how many kilos (or bundles) are sold fresh and processed ….				Selling Price
	1 back reef / lagoon<br>2 fore reef slope<br>3 reef channel<br>4 mangrove<br>5 seagrass<br>6 deep water<br>7 other	1. line fishing<br>2 free diving<br>3 net fishing	1. Regularly – each week<br><br>2. Occasionally	(G)<br><br>Maximum catch (kg)	(B)<br><br>Minimum catch (kg)	(N)<br><br>Normal catch (kg)	Food	Share	Trade	Dried, salted or smoked (D, S, Sm)	Sold fresh	To the local people (kg)		To a vendor (kg)		**Specify Units**
												Fresh	D, S, Sm	Fresh	D, S, Sm	Per kg or per unit
01 Reef fish																
02 Small pelagics																
03 Coastal pelagic																
04 Tuna																
05 Ocean pelagic																
06 Ray																
07 Shark																
08 Squid																
09 Octopus																
10 Sea cucumber																
11 Lobster																
12 Shrimp																
13 Crabs																
14 Other																

FISHER ONLY-TABLE 2 - REEF FISH DETAIL: Daily catch on good (G), bad (B) or normal (N) days (one row per habitat type). Add more rows for other main families / key species

Reef Fish type	Fishing site	Type of fishing	How many kg (or bundles) is your maximum, minimum and average daily catch in each reef fish type?			In the total catch, what proportion (%) or how many kilos / bundles are for:					For the catch sold, how many kilos / bundles are sold fresh and processed (D, S, Sm)?				Selling price
	1 back reef / lagoon<br>2 fore reef slope<br>3 reef channel<br>4 mangrove<br>5 seagrass<br>6 deep water<br>7 other	1. line fishing<br>2 free diving<br>3 net fishing	Maximum catch<br><br>(G)	Minimum catch<br><br>(B)	Normal catch<br><br>(N)	food	Share	trade	Dried, salted or smoked	Selling fresh	To local people (kg)		To a vendor (kg)		
											Fresh	D, S, Sm	Fresh	D, S, Sm	Per kg or unit (specify)
01 Parrotfish															
02 Emperor															
03 Surgeonfish															
04 Grouper 1 (kawakawa)															
05 Grouper 2 (donu)															
06 Rabbitfish															
07 Snapper															
08 Sweetlips															
09 Wrasse															
10 Triggerfish															
11. Unicornfish															
12. Goatfish															
13. Other (mullet?)															

Per trip, do you target particular types of reef fish or take whatever you can catch? : Target / Not Target **(circle one**)

If targeting then list the Reef Fish Types targeted (Use table on last page/fish guides):

GLEANER ONLY-TABLE 3: When you go gleaning, estimate all the catch for one day on a good (G), bad (B) or normal (N) day (**one row per habitat type - use extra pages)**

	habitat	Glean on spring tide or neap tide?	Around how many kilos (or units) is the maximum, minimum and your normal catch				In the total catch, what proportion (%) or how many kilos are for					For the catch that you sell, how many kilos are selling fresh and processed (dried, salted or smoked) ?				Selling price
	1 back reef / lagoon<br>2 mud<br>3 rocks<br>4 mangrove<br>5 seagrass<br>6 other	1 spring tide<br>2 neap tide	1. kg<br>2. unit	Maximum catch<br>(G)	Minimum catch<br>(B)	Normal catch<br>(N)	food	Share	trade	Dried, salted or smoked	Sold fresh	To local people		To a vendor		
												Fresh	Dried, salted, or smoked	Fresh	Dried, salted or smoked	Per kg or unit - specify
01 Octopus																
02 Sea cucumber																
03 Trochus																
04 Crab																
05 Urchin																
06 Giant clams																
07 Other molluscs																
Other (specify)																

FISHER AND GLEANER TABLE 4: When you are fishing or gleaning ____ days per 2 weeks, how many days get you a good catch, bad catch, medium catch?

Weather	Number of days for fishing	Number of days that the catch is good	Number of days that the catch is bad	Number of days that the catch is normal
01 Good				
02 Bad				
	Number of days for gleaning	Number of days that the catch is good	Number of days that the catch is bad	Number of days that the catch is normal
03 Good				
04 Bad				

SECTION III : HOUSEHOLD CONSUMPTION

Do you normally eat fish or marine products in your household?

☐ Yes ☐ No ☐ Only on special occasions

How important is fresh fish consumption to your household?

☐ Most ☐ Very ☐ Important ☐ Neutral ☐ Little ☐ Least ☐ No importance

How many days per week do you normally eat fresh fish? (Between 1 and 7) :________________________

How many days per week do you normally eat tinned fish? (Between 1 and 7) :_______________________

How many days per week do you normally eat beef? (Between 1 and 7) : ____________________________

How many days per week do you normally eat chicken? (Between 1 and 7) : _________________________

How many days per week do you normally eat pork? (Between 1 and 7) :_____________________________

How many days per week do you normally eat tinned meat? (Between 1 and 7) : ______________________

What is your primary source of fresh fish? (select one)

☐ Bought ☐ own catch ☐ traded / barter ☐ Gifted ☐ Other

What type of fish/seafood do you normally eat? (can select more than one)

☐ Reef fish ☐ Coastal Pelagic ☐ Tuna ☐ Ocean Pelagic ☐ Small pelagic ☐ Shellfish ☐ Other (specify)

NOTES

Fish Groupings Definitions :

Small Pelagic fish	Scads, small mackerel (Salala), sardines, garfish (busa)
Coastal pelagic fish	Trevally (Saqa), spanish mackerel (walu), barracuda (Ogo)
Ocean pelagic fish	Mahimahi (Maimai/Saqakula/Dolofini), wahoo (Wau), sailfish (Sakuvorowaqa), marlin (Saku), or similar
Tuna	Skipjack, yellowfin or similar (Yatu)

TABLE OF FISH / INVERTEBRATE NAMES IN ENGLISH AND FIJIAN

Fin-fish			Invertebrates	
**English Name (Family)**	**Species - latin (common name)**	**Fijian Name**	**English Name**	**Fijian Name**
Parrotfish 1 (smaller parrotfish)	Chlorurus bleekeri, Scarus rubroviolaceus, S. globiceps, S. ghobban, S. niger, S. rivulatus	Rawarawa	Octopus	Kuita
Parrotfish 2	Hipposcarus longiceps, Scarus.rubroviolaceus, S. ghobban	Ulavi	Sea cucumber	Sasalu/Dairo
Emperor 1 (smaller, long body shape, slanting snout)	Lethrinus harak (Thumbprint)<br>(L. rubrioperculatus, L. semicinctus)	Kabatia	Trochus<br>Common turban shell	Sici<br>Tovu/La
Emperor 2 (larger, deeper body, more vertical snout)	Lethrinus atkinsoni (Pacific yellowtail)<br>L. nebulosus (Spangled)<br>L. xanthochilus (Yellowlip)<br>L. olivaceous (Longface)	Sabutu<br>Kawago<br>Kacika<br>Dokonivudi	Crab	Qari
Surgeonfish	Ctenochaetus striatus (Striated), Acanthurus lineatus (Lined)	Meto or Ikaloa or Dridri / Dridrinitoga / Balagi	Urchin	Cawaki
Grouper 1 (camouflage spp.)	Epinephelus spp.	Kawakawa	Other shells / molluscs	
Grouper 2 (rock cod / coral trout)	Plectropomus spp.	Droudroua / Donu	Smooth giant clam / giant clam / rugose giant clam / fluted giant clam	Vasuadina / vasua matau / katavatu / vasua cega
Rabbitfish	Siganus vermiculatus, S. doliatus	Nuqa / Nuqanuqa		
Snapper 1 (smaller bodied)	Lutjanus fulvus (Blacktail), L. semicinctus (Halfbarred).	Kake		
Snapper 2 (larger bodied)	Lutjanus bohar (twospot red),<br>L. argentimaculatus (mangrove jack)<br>Macolor niger (black and white)	Bati / Baji<br>Damu<br>Guruniwai		
Sweetlips	Plectorhinchus spp.	Sevaseva / Drekeni		
Wrasse	Cheilinus trilobatus (Tripletail) / C. undulatus (Humphead)	Draunikura / Draudrau or Varivoce		
Triggerfish	Any species	Cumu		
Unicornfish	Naso sp.	Ta		
Goatfish	Parupeneus sp.	Ose / Osekula / Ki		
Mullet	Crenimugil crenalabis, Liza vaigiensis, Mugil cephalus	Kanace / Keteleka / Kava / Koto		

Appendix S2. Marine Resource Catch and Monetary Value Estimation

A- Adjusting for seasons and weather conditions

The range of questions asked in the survey (Appendix S1, section 2) regarding fishing and gleaning in different seasons and weather conditions is designed to ensure that the study is representative of fishing conditions on Kadavu for a full calendar year. The number of good/bad weather days was taken into account for the catch estimates by calculating the mean number of days per month that had good or bad weather according to the fishers' or gleaners' responses on weather. The catch responses from fishers or gleaners were then adjusted to provide a more realistic estimate of actual days spent fishing or gleaning per month. This was done by adjusting the 'number of days fishing or gleaning' responses of individual fishers and gleaners for each weather and season combination by the estimated proportion of days per month that had good or bad weather in each season. The catch estimates were next adjusted for the probability (as a proportion of 1) of having a good, normal, or bad catch in either good or bad weather. This probability was estimated by calculating the averages from the responses provided for Table 4 of the questionnaire (Appendix S1, section 2). Factoring in the range of catch (maximum, average, minimum) and the effect of weather and season is a standard method to reduce bias in interview-derived data (O'Donnell et al. 2012).

B- Finfish and invertebrate categories

Finfish categories included: reef fish (twelve families along with an 'other' category), four types of pelagic fish (small pelagic, coastal pelagic, offshore pelagic, and tuna), and elasmobranchs (sharks and rays). For reef fish, fishers were also asked if they targeted particular families when fishing. Invertebrate taxa harvested by fishers included: molluscs (giant clam, reef squid, and octopus), crustaceans (lobster, crab, and shrimp), and holothurians (sea cucumbers). The main habitats where each category of finfish was caught were recorded using the following categories: back reef / lagoon, fore reef slope, reef channel, mangrove, seagrass, deep water or 'other'. Habitat categories for gleaning differed with 'mud' and 'rocks' replacing fore reef slope, reef channel, and deep water. Gleaners were asked about catches for sea urchin, sea cucumber, crab, four categories of mollusc (octopus, trochus, giant clam, and 'other molluscs'), and 'other'.

C- Conversion of catch records to weight and value

The weights of finfish and invertebrates were derived from a combination of stated sizes (length) and/or weights (in kg or bundles[1]). For finfish, the mean sizes of reef or pelagic fish were calculated using both the information collected from interviewees for unsold fish, and fish size data collected in Kavala Bay for sold fish as part of the TAILS monitoring programme[2] in 2017-2018. These mean sizes were used to convert catch data for individual fish into weight (Table S2), using the length-weight (L/W) relationships available for reef or pelagic fish genera or species on Fishbase (www.fishbase.in). Regional estimates for the L/W relationships were used where available on Fishbase. Weights were calculated separately for sold and unsold fish as semi-commercial fishers were generally selling the larger fish in their daily catch and keeping the smaller ones for food.

Catch records for reef fish families were analysed to determine relative fishing pressure at the family level. For some fishers, the sum of the responses for individual reef fish families was greater than the

[1] Where 1 bundle of fish = 4 kg.

[2] Collaborative data collection programme for coastal fisheries between the Pacific Community (SPC) and Fiji's Ministry of Fisheries.

single response for reef fish total catch. It was assumed that some fishers had over-estimated the daily catch when responses were recorded for individual reef fish families. Therefore, to assess relative fishing pressure on reef fish families, the proportion of each family caught by fishers on a daily basis was calculated for fishers who provided responses as catch weights (bundles or kg). Catch proportions were calculated for the different fishing methods and for all types of fishing combined.

In a few cases, only the number of individuals for the total reef fish catch were recorded. For these data, the responses provided for reef fish families were used to calculate the proportion of the total catch per family. These proportions were then used to estimate the weight of the total reef fish catch (using the mean lengths for sold or unsold fish at the family or genus level). The average of the sold and unsold lengths were used for some calculations for fishers who primarily fished commercially but kept a proportion of their catch for local consumption as these fishers were targeting a range of fish sizes.

After comparing the responses for total reef fish caught to the sum of the results for reef fish families it was decided to use the total reef fish responses to calculate annual estimates of catch and value. The reasoning for this was that some fishers overestimated the catch for reef fish families so that when the records for individual families were summed up they substantially exceeded the single response for total reef fish catch. The latter was therefore regarded as the more accurate measure of reef fish catch.

Catch estimates were converted to monthly amounts, seasonal amounts (6 months each for cyclone and non-cyclone seasons), and finally an annual catch estimate. Occasional catches were factored in by asking key informant fishers how often they would catch categories that were harvested more occasionally, e.g. once in five trips for coastal pelagic fish. Seasonal catches were included where recorded for specific parts of the year, e.g. six out of 12 months for tuna.

The extrapolation of catch data to the district level was based on the mean catches of individual fishers or gleaners multiplied by the mean number of people per household practicing each type of marine resource collection (fishing, fishing and gleaning, or gleaning only). This was then multiplied by the number of households in the district.

Catch estimates were converted to monetary value based on unpublished local values for sold or unsold (retained) finfish or invertebrates using the prices charged by fishers or gleaners on Kadavu. These selling prices were collected either during the household survey or from key informants for the same time period that the surveys were conducted in 2019 (see Table S1). For reef fish, the values used were set as a standard of $25 per bundle when sold commercially. The selling price did vary according to point of sale and whether the fisher was mainly fishing commercially or occasionally sold some of the catch. For example, the selling price within a community was generally less than that charged by commercial fishers e.g. $10-20 per bundle rather than $25. Fish prices did not change between seasons but were affected by other factors such as the knock-on effects of the COVID-19 lockdown in 2020 where fishers from Nakasaleka (Matasawalevu) travelled to Vunisea rather than Kavala to sell their catch when the inter-island ferry service was postponed. The longer travel distances by boat resulted in a price of $35 per bundle rather than $25 to cover the increased fuel costs. It is not known if Kadavu fish prices were published in annual reports of the Ministry of Fisheries but records of local fish prices were kept at the ministry's fisheries stations on Kadavu in Vunisea and Kavala.

References

O'Donnell, K.P., P.P. Molloy, and A.C.J. Vincent. 2012. Comparing fisher interviews, logbooks and catch landings estimates of extraction rates in a small-scale fishery. *Coastal Management* 40: 594–611.

Table S1 Marine Resource Category Prices used in the calculation of Total Value

Marine Resource Category	Price per kg (FJD)	Notes
Reef Fish	6.25	$25 / bundle
Small Pelagic	6.25	$25 / bundle
Coastal Pelagic	6.25	$25 / bundle
Tuna	6	Set price paid at Fisheries Stations on Kadavu
Ocean Pelagic	6	
Sharks and Rays	6	
Squid	20	Small individuals: $10 (0.5 kg)
Octopus	12	Small and medium octopus prices combined
Holothurians	10	Wet weight
Lobster	21	
Crab (Mangrove)	20	
Crab (Land)	33.3	*Cardisoma cardifex*
Trochus (shell)	7.25	Mean (n = 4)
Trochus (meat)	5	Shell to meat ratio of 5:1
Giant Clam (meat)	10	Shell to meat ratio of 5:1
Mixed Shells (meat)	2	Shell to meat ratio of 5:1

Table S2. Mean Size (Total Length) of the main reef fish harvested and conversion to weight

Fijian name	Common name	Genera / Species	Sold	Sold	Not Sold	Not Sold	Sold / not sold combined	
			L (cm)	W (g)	L (cm)	W (g)	L (cm)	W (g)
Sabutu	Pacific yellowtail emperor	*Lethrinus atkinsoni*	32.62	751.48	18.96	145.13	25.79	368.75
Kabatia	Blackspot emperor	*Lethrinus harak*	29.29	506.60	17.66	110.46	23.47	260.18
Ulavi	Parrotfish (smaller bodied)	*Scarus / Hipposcarus spp.*	39.55	1325.00	24.72	318.63	32.13	705.66
Ulurua	Steephead parrotfish	*Chlororus microrhinus*	43.28	1836.49	(25.00)	340.58	34.14	886.48
Donu	Coral grouper	*Plectropomus spp.*	42.11	1318.79	28.44	388.86	35.28	760.33
Kawakawa	Camouflage grouper	*Epinephelus spp.*	40.35	1082.65	31.07	483.65	35.71	742.88
Ta	Bluespine unicornfish	*Naso unicornus*	45.43	2117.58	32.50	793.67	38.97	1351.02
Balagi	Surgeonfish	*Acanthurus spp.*	39.72	1434.90	27.5	494.19	33.61	884.27
Mama	(Big-eye) Bream	*Gymnocranius / Monotaxis spp.*	35.12	677.93	22.50	193.05	28.81	387.75
Dokonivudi	Longface emperor	*Lethrinus olivaceus*	45.16	1387.11	(30.00)	467.60	37.58	815.57
Kake	Snapper (smaller bodied)	*Lutjanus spp.*	(30.00)	408.34	18.75	97.84	24.38	217.35
Bo	Paddletail snapper	*Lutjanus gibbus*	32.61	662.11	18.75	125.90	25.68	323.46
Sevaseva	Sweetlips	*Plectorhincus spp.*	41.77	1837.27	27.50	520.00	34.63	1043.19
Kacika	Yellowlip emperor	*Lethrinus xanthochilus*	42.13	1313.78	20.00	144.77	31.07	533.29
Matalevu	Soldierfish	*Myriprisitis spp. (berndti)*	36.32	1136.22	18.75	164.76	27.54	506.27
Ose	Goatfish	*Mulloidichthys / Parupeneus spp.*	34.09	657.08	16.00	66.03	25.04	257.38
Nuqa	Rabbitfish	*Siganus spp.*	27.49	500.10	20.00	188.45	23.74	318.98
Cumu	Triggerfish	*Sufflamen / Balistoides / Rhinecanthus spp.*	n.d	n.d	14.96	95.40	n.d	n.d
Draunikura	(tripletail) wrasse	*Cheilinus spp. (trilobatus)*	15.00	78.03	13.57	57.67	14.29	67.40
Kava	mullet	*Ellochelon vaigiensis*	n.d	n.d	24.50	216.12	n.d	n.d
Senikawakawa	Honeycomb grouper	*Epinephelus merra*	n.d	n.d	14.13	42.54	n.d	n.d
Kawago	Spangled emperor	*Lethrinus nebulosus*	41.79	1402.21	(30.00)	543.39	35.90	907.70
Damu	Mangrove snapper	*Lutjanus argentimaculatus*	n.d	n.d	(30.00)	482.31	n.d	n.d
Kanace	mullet	*Crenimugil spp.*	n.d	n.d	27.50	306.61	n.d	n.d
Sabutudamu	Yellowspotted emperor	*Lethrinus kallopterus*	41.95	1423.03	(35.00)	820.49	38.48	1094.09
Ikaloa	Surgeonfish (smaller)	*Acanthurus / Ctenochaetus spp.*	n.d	n.d	18.13	151.07	n.d	n.d

Where: L = length, W = weight, cm = centimetres, g = grams, n.d. = no data.
Lengths in brackets were estimates based on discussions with key local informants.
